# Fluorescence Studies on the Binding Affinity and Determination of Vitamin B12 in the Presence of Fibrinogen

**DOI:** 10.1007/s10895-024-03835-1

**Published:** 2024-07-15

**Authors:** Elmas Gökoğlu, Seniye Şura Budun, Bensu Doyuran, Tugba Taskin-Tok

**Affiliations:** 1 Faculty of Science, Department of Chemistry, Hacettepe University, Ankara, 06800 Turkey; 2 Faculty of Arts and Sciences, Department of Chemistry, Gaziantep University, Gaziantep, 27310 Turkey; 3 Department of Bioinformatics and Computational Biology, Gaziantep University, Gaziantep, 27310 Turkey

**Keywords:** Fibrinogen, Vitamin B12, Fluorescence spectroscopy, Molecular docking, FRET

## Abstract

The binding properties between vitamin B12 (vitB12, cyanocobalamin) and fibrinogen (Fib) were investigated by UV-vis absorption and steady-state/three-dimentional (3D) fluorescence spectra techniques as well as molecular docking. The experimental results showed that the intrinsic fluorescence of Fib quenched by vitB12 with static mechanism to form a non-fluorescent complex. The positive signs of thermodynamic parameters, ΔH (92.18 kJ/mol) and ΔS (433.5 J/molK), indicated that the hydrophobic forces were dominant in the binding mode. The molecular docking data were found to be in agreement with these experimental results and were confirmed by three hydrophobic interactions between the Trp430, Try390 residues of Fib and the vitamin. 3D spectra showed that fibrinogen undergoes a conformation change when it interacts with vitB12. Based on non-radiative energy transfer theory, binding distance was calculated to be 3.94 nm between donor (tryptophan residues of Fib) and acceptor (vitB12). The limit of detection (LOD) of vitB12 was calculated as 2.08 µM in the presence of fibrinogen. The relative standard deviation (RSD) of method was 4.28% for determinations (*n* = 7) of a vitB12 solution with the concentration of 7.80 µM.

## Introduction

Vitamin B12 (cyanocobalamin) has a tetrapyrrole ring that contains a Co atom in its structure. It is naturally found in dairy product, meat, egg, fish and shellfish. It is an important water-soluble vitamin and plays several roles in human physiology such as red blood cell formation, brain and nerve functions, and its deficiency leads to weight loss, anemia, weakness, nausea, constipation and neuropaty [1, 2]. VitB12 is needed to keep the blood healthy and is used by the body to make proteins. The determination of vitB12 is very important to find the deficiency of vitamin in the body and to identify its amount in various foods and microbial environments. For this, sensitive analytical methods are needed [3]. Fibrinogen is a fibrillar protein with a molecular mass of 340 kDa, composed of three double chains forming the molecular structure (AαBβγ)_2_ [4]. Its dimeric structure consists of three non-identical Aα-, Bβ- and γ-chain subunits connected by 29 disulfide bonds. The subunits take the form of a central nodule (E-region) and two identical outer nodules (D-region) connected by three helical α-helical coils. It has a total of 72 intrinsically fluorescent trptophan residues in its primary structure. Their distribution within the structure is as follows: (*α* = 11; *β* = 14; *γ* = 11)_2_. The part of the blood plasma of vertebrates, the main function of fibrinogen is to form fibrin clots to prevent blood loss due to injury [5]. It has several biological functions including triggering hemostasis, fibrinolysis, facilitating platelet adhesion and aggregation, activating leukocytes, cellular and matrix interactions and wound healing [6]. The studies of the fluorescence quenching of protein by a small molecule such as drug, dye, flourescent probe, metal complex etc. are important to explain binding properties of protein with the molecule. There are some reports to publish on the interactions protein with vitB12 by fluorescence quenching such as lysozyme [7], BSA [8], HSA [9], hemoglobin [10]. There is no study on the interaction of fibrinogen, an important protein in blood clotting, and vitB12 which is very important for blood health. The interactions between vitB12 and fibrinogen can be predicted to have important effects on a wide range of issues, from blood clotting to cardiovascular health, neurological function and nutritional strategies. Here, binding affinity of B12 with fibrinogen was studied by steady-state and 3D-fluorescence; Uv-vis absorption and molecular docking methods. The binding parameters, thermodynamic analysis, intermolecular distance were investigated of vitB12-Fib system. Moreover, determination of vitB12 in the presence of fibrinogen using fluorescence quenching method was given with LOD and LOQ values.

## Experimental

### Chemicals

Fibrinogen (Fib, from porcine plasma, MW: 340 kDa) and vitamin B12 (vitB12, cyanocobalamin) were purchased from Sigma-Aldrich (Chem. Co., USA). All reagents were used of analytical grade and without purification. Experimental studies were carried out in PBS (phosphate buffer solution) at pH 7.4 buffer solution media prepared with double distilled water. The solutions used in experimental studies were prepared by diluting stock solutions with PBS and stored at 4 ^o^C.

### Instruments and Procedures

#### Fluorescence Studies

A F-4500 Spectrofluorometer (Hitachi, Japan) containing 150 W Xe lamp and FLSolutions software was used for all fluorescence measurements. The steady-state fluorescence experiments were performed on band slits for both excitation and emission at 2.5 nm with scan speed 240 nm/min. The fluorimetric titrations were performed manually using a 1.0 mM stock solution of vitB12 which was dissolved in PBS at pH 7.4. The fibrinogen stock solution was prepared in same PBS at the concentration equal to 10 µM. Trp residues of fibrinogen was excited at 280 nm all fluorescence experiments. Each step during titration of 5 µL stock solution of vitB12 was added to 2500 µL of 1.0 µM fibrinogen solution by microliter injections. Final titration solution in a cell was 2550 µL. The concentration of fibrinogen during interacting with vitB12 was changing in the range 1.0-0.98 µM. The final concentration of vitB12 in titration solution was 19.3 µM. The titration assays were repeated at three temperatures (298, 303 and 310 K) and results were plotted according to Stern-Volmer equation, and also the quenching and binding constants; thermodynamic parameters and also according to Förster theory, fluorescence resonance energy transfer (FRET) parameters were calculated and explained using the titration data. Here, the corrected fluorescence intensities were used in all data based on the inner filter effect [11].

The three-dimensional (3D) fluorescence spectra were obtained under the following conditions; the exitation and emission wavelengths ranged from 200 to 450 nm, band slits at 5 nm, interval for excitation and emission wavelength at 10 nm with scan speed 1200 nm/min. A Mettler Toledo (Five Easy Plus) digital pHmeter was used for adjusting pH of solutions.

### UV Absorbance Studies

UV-vis absorption spectra were recorded with an UV-1700 PharmaSpec (Shimadzu, Japan) spectrophotometer containing UVProbe software. All measurements were performed in a 1 cm quartz cell in PBS at pH 7.4 at room temperature. In experiments, fixed concentration of fibrinogen (1.0 µM) was titrated with the successive addition of vitB12 (0-7.6 µM).

### Molecular Docking Studies

AutoDock Vina [12, 13] was applied to obtain important information about the interaction mechanisms of vitB12-Fib system for molecular docking process. First, compound vitB12 as ligand and fibrinogen (Sigma, F2629, from porcine plasma), as target were generated by using homology modelling method. Then this model was organized by Discovery Studio (DS) 3.5 [14] before docking process. The geometry and energy optimization of the relevant compound and target model were performed using the CHARMm force field and the adopted basic Newton-Raphson (ABNR) method [15]. Available in the DS 3.5 protocol until the mean square deviation (RMSD) gradient is < 0.05 kcal/mol Å^2^. To define the active site(s) of the fibrinogen, related literature and also the DS tools were used. End of these steps, AutoDock Vina was exerted using default settings. 200 conformational images were further generated for fibrinogen-interacting compound based on the Lamarckian genetic algorithm. Docking findings, including the best pose, docking energy, interaction types and RMSD value, were evaluated for the compound vitB12 with the lowest binding energy in the target structure.

## Results and Discussion

### Fluorescence Quenching Measurements

**Fig. 1:**
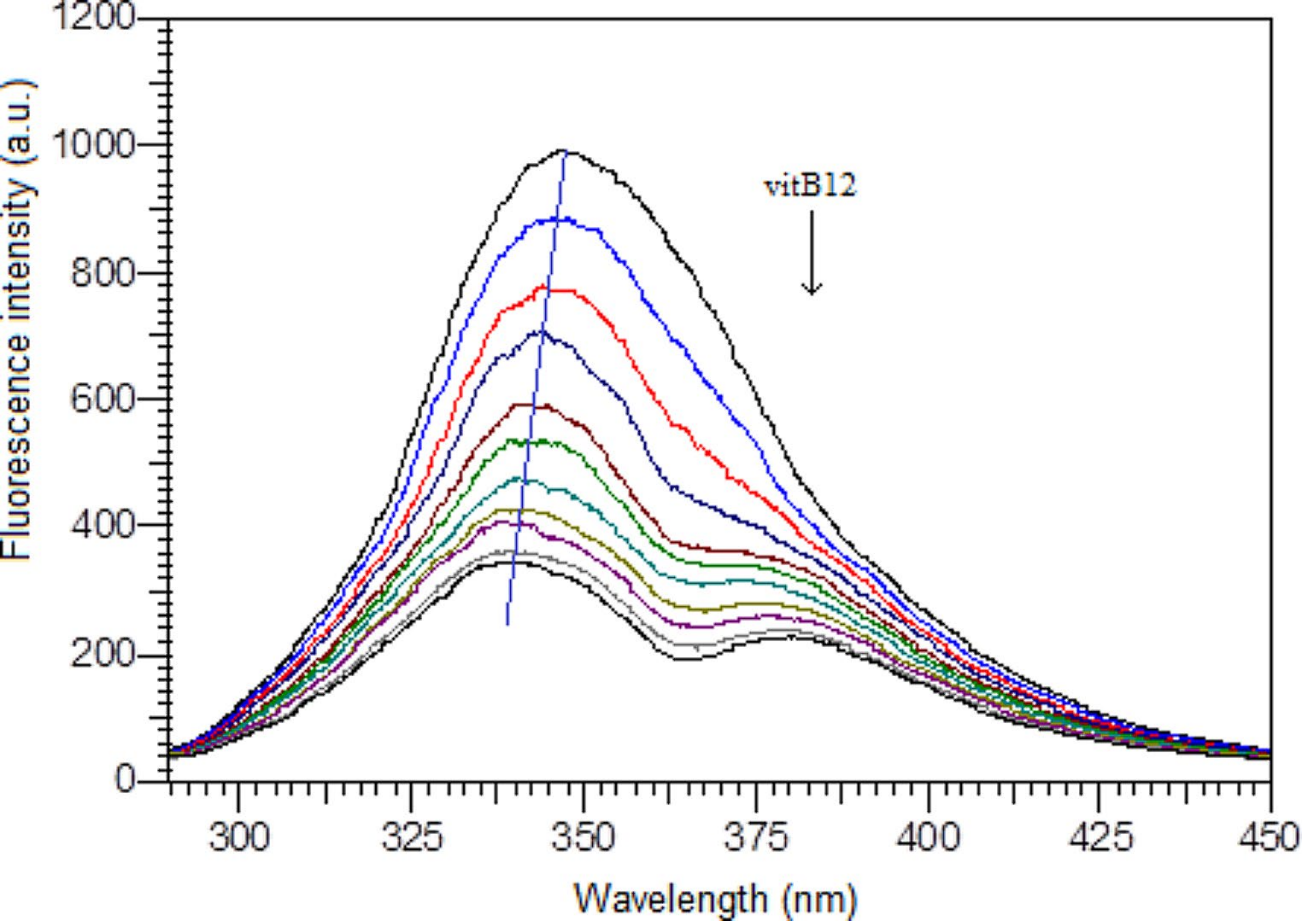
Effect of vitB12 on fluorescence spectra of Fib. c(Fib) = 1.0 µM, c(vitB12): 0, 1.96; 3,92; 5.86; 7.80; 9.73, 11.7; 13.6; 15.5; 17.4; 19.3 µM, conditions: pH 7.4, 298 K, λ_ex_ = 280 nm

The fluorescence spectroscopic methods are widely used to elucidate the interactions between biomolecules and various species such as fluorescent probe, drug, metal or metal complex, dye, etc. The decrease in fluorescence intensity in the presence of a quencher points a variety of processes; collisional quenching, molecular rearrengement, excited state reaction and also the ground state complex formation [11]. The quenching of fluorescence can be divided into dynamic and/or static mechanisms which distinquishe by their temperature and viscosity dependence. The gradual addition of vitB12 into the constant concentration of Fib results in decrease in fluorescence intensity of Trp residues of Fib molecule. Figure 1 shows the fluorescence emission spectra of Fib with various amounts of vitB12 following on excitation wavelength at 280 nm. Under the same conditions, there is no emission of vitB12 at the given wavelength range. The blue-shift in the maximum emission wavelength from 348 nm to 337 nm suggests an increased hydrophobicity of the region surrounding of fibrinogen fluorescent residues [6]. The quenching of fluorescence was described using Stern-Volmer equation [11].

1 $${{\rm{F}}_{\rm{0}}}{\rm{/F}}\,{\rm{ = }}\,{\rm{1}}\,{\rm{ + }}\,{{\rm{K}}_{{\rm{sv}}}}\left[ {\rm{Q}} \right]\,{\rm{ = }}\,{\rm{1}}\,{\rm{ + }}\,{{\rm{k}}_{\rm{q}}}\,{{\rm{t}}_{\rm{0}}}\left[ {\rm{Q}} \right]$$

where F_0_ and F show the Trp fluorescence intensities from Fib in absence and presence of quencher (vitB12), respectively. Ksv shows Stern-Volmer quenching constant, [Q] indicates the quencher concentration. The magnitude of K_sv_ can be obtained by linear regression of F_0_/F data against [Q]. k_q_ and τ_0_, are the biomolecular quenching constant and the average biomolecule lifetime without quencher, respectively. k_q_ is calculated from K_sv_/τ_0_ ratio using τ_0_ = 10^− 8^ s for biomolecules [16].

**Fig. 2:**
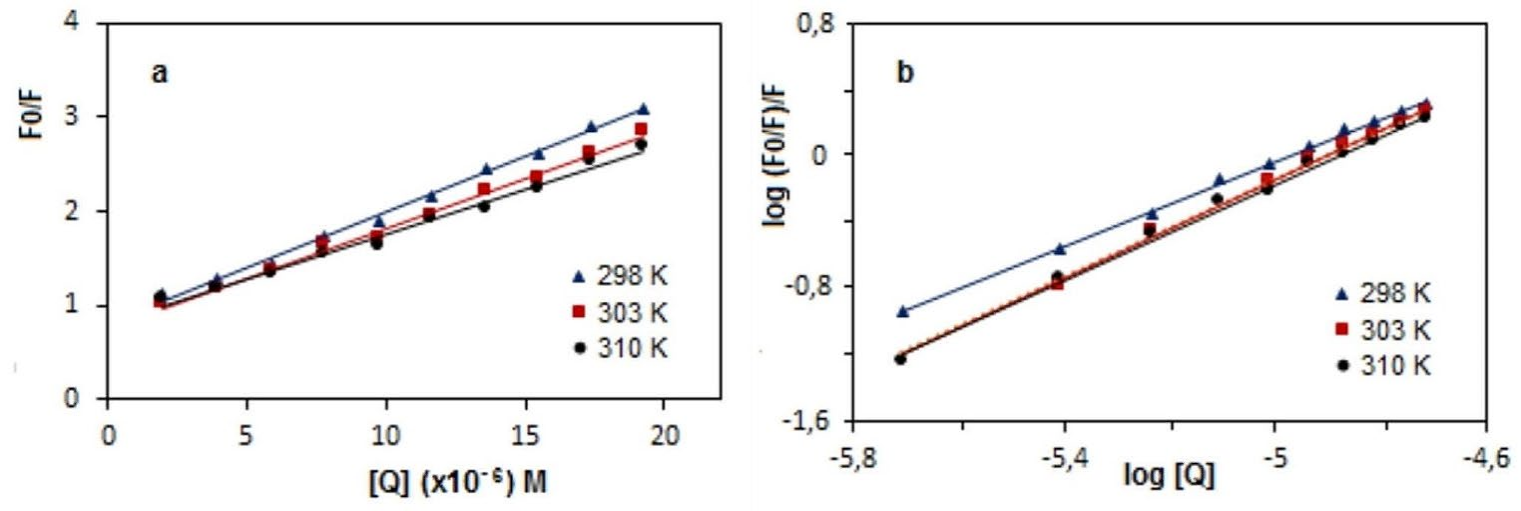
Stern-Volmer plots **(a)** and double log plots **(b)** of vitB12 and Fib system at three temperatures

Figure 2a gives a good linear Stern-Volmer plots at three temperatures and calculated K_sv_ and k_q_ values are listed in Table 1. k_q_ value was obtained in the range of 11.85–9.55 × 10^12^ M^− 1^ s^− 1^ at three temperature. The limit value of k_q_ for dynamic quenching in interaction system between small molecule and biomolecule is 1.0 × 10^10^ M^− 1^ s^− 1^ [16, 17]. The fact that the k_q_ values are much higher than rate constant of maximum scatter collision-quenching indicates the possible quenching mechanism is *static.* This shows that a complex has formed between vitB12 and fibrinogen. Also, as another evidence for *static* quenching is that K_sv_ values of the system decrease with increasing temperature.

For the static quenching process in protein-small molecule interaction, the binding constant (K_a_) and the number of binding sites (n) can be obtained from the following equation [18].

2 $${\rm{log}}\left( {{{\rm{F}}_{\rm{0}}}\,{\rm{-}}\,{\rm{F}}} \right){\rm{/F}}\,{\rm{ = }}\,{\rm{log}}\,{{\rm{K}}_{\rm{a}}}\,{\rm{ + }}\,{\rm{n}}\,{\rm{log}}\left[ {\rm{Q}} \right]$$

where F_0_, F and [Q] are the same as in Eq. (1). The obtained double-log plots are shown in Fig. 2b and the corresponding values are given in Table 1. The n values are approximately equaled to one, showing that there is one binding site for vitB12 on the Fib molecule. K_a_ constants increases with increase in temperature suggesting that vitB12-Fib complex has more stable and high binding capacity at high temperature.

**Table 1:** Stern-Volmer quenching constants; binding constants, binding site numbers and thermodynamic parameters for vitB12-Fib system

T (K)	K_sv_	k_q_	K_a_	*n*	ΔS	ΔH	ΔG
	(x10^4^ M^-1^)	(x10^12^ M^-1^s^-1^)	(x10^6^ M^-1^)		(kjmol^-1^)	(kjmol^-1^)	(jmol^-1^K^-1^)
298	11.82	11.82	2.818	1.29	-37.00	92.18	433.5
303	10.57	10.57	6.486	1.39	-39.17		
310	9.55	9.55	12.13	1.45	-42.20		

### Binding Forces Between vitB12 and Fibrinogen

**Fig. 3:**
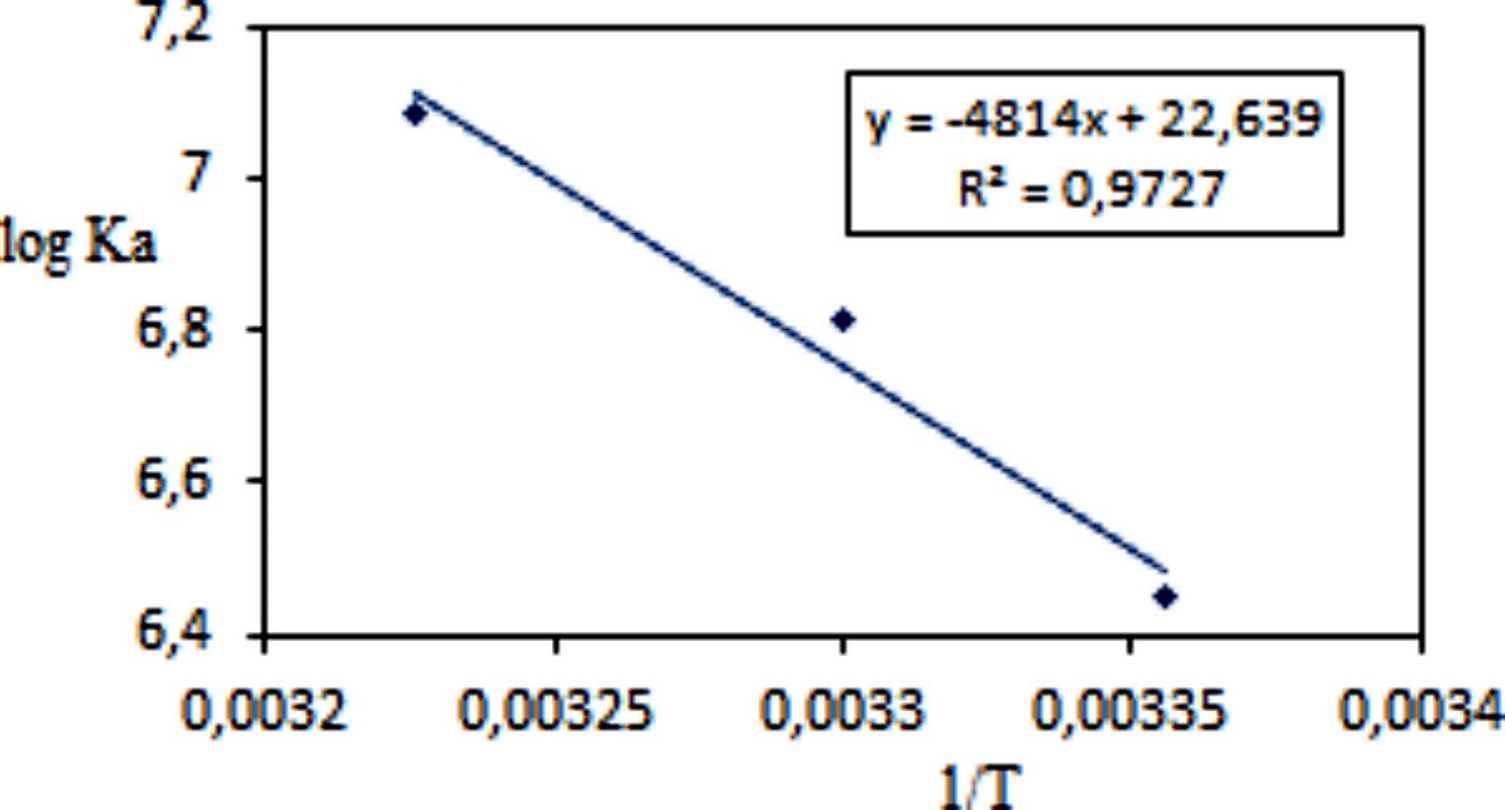
van’t Hoff plot for the vitB12-Fib system

The fluorescence quenching is widely used in studying the binding modes between protein and small molecules. Ross and Subramanian [19] explained the thermodynamic data of judging the primary binding acting force of protein with the molecule; (i) ΔH < 0 and ΔS < 0 indicate that van der Waals force and hydrogen bonding are the main forces, (ii) ΔH = 0 and ΔS > 0 suggest electrostatic interactions, (iii) ΔH > 0 and ΔS > 0 show a hydrophobic interaction. The enthalpy change (ΔH) and entropy change (ΔS) parameters can be obtained via the van’t Hoff equation;

3 $${\rm{log}}\,{{\rm{K}}_{\rm{a}}}\,{\rm{ = }}\, - \Delta {\rm{H/2}}{\rm{.303}}\,{\rm{RT}}\,{\rm{ + }}\,\Delta {\rm{S/2}}{\rm{.303}}\,{\rm{R }}$$

where K_b_ and R are binding constant and gas constant, respectively. It can be seen from Fig. 3 that there is a linear relationship between log K_a_ and 1/T in plot with the equation log K_a_ = − 4814 1/T + 22.639 (R^2^ = 0.9727). Gibbs free energy change (ΔG) was calculated from Eq. (4);

4 $$\Delta {\rm{G}}\,{\rm{ = }}\,\Delta {\rm{H}}\,{\rm{-}}\,{\rm{T}}\Delta {\rm{S}}\,{\rm{ = }}\, - \,{\rm{RTln}}{{\rm{K}}_{\rm{a}}}$$

ΔH could be obtained from the slope of plot, whereas ΔS can be calculated from the intercept. All thermodynamic data are listed in Table 1. The negative ΔG and posivite ΔH showed the spontaneous and endothermic binding process. The both positive value of ΔH (92.18 kjmol^− 1^) and ΔS (433.5 jmol^− 1^K^− 1^) suggested that the acting forces were mainly hydrophobic interactions in binding between vitB12 and Fib. This is consistent with the molecular docking results, and a total of three hydrophobic interactions were identified in the binding involving Try and Trp residues of Fib in the relevant section.

### Energy Transfer from Fibrinogen to vitB12

**Fig. 4:**
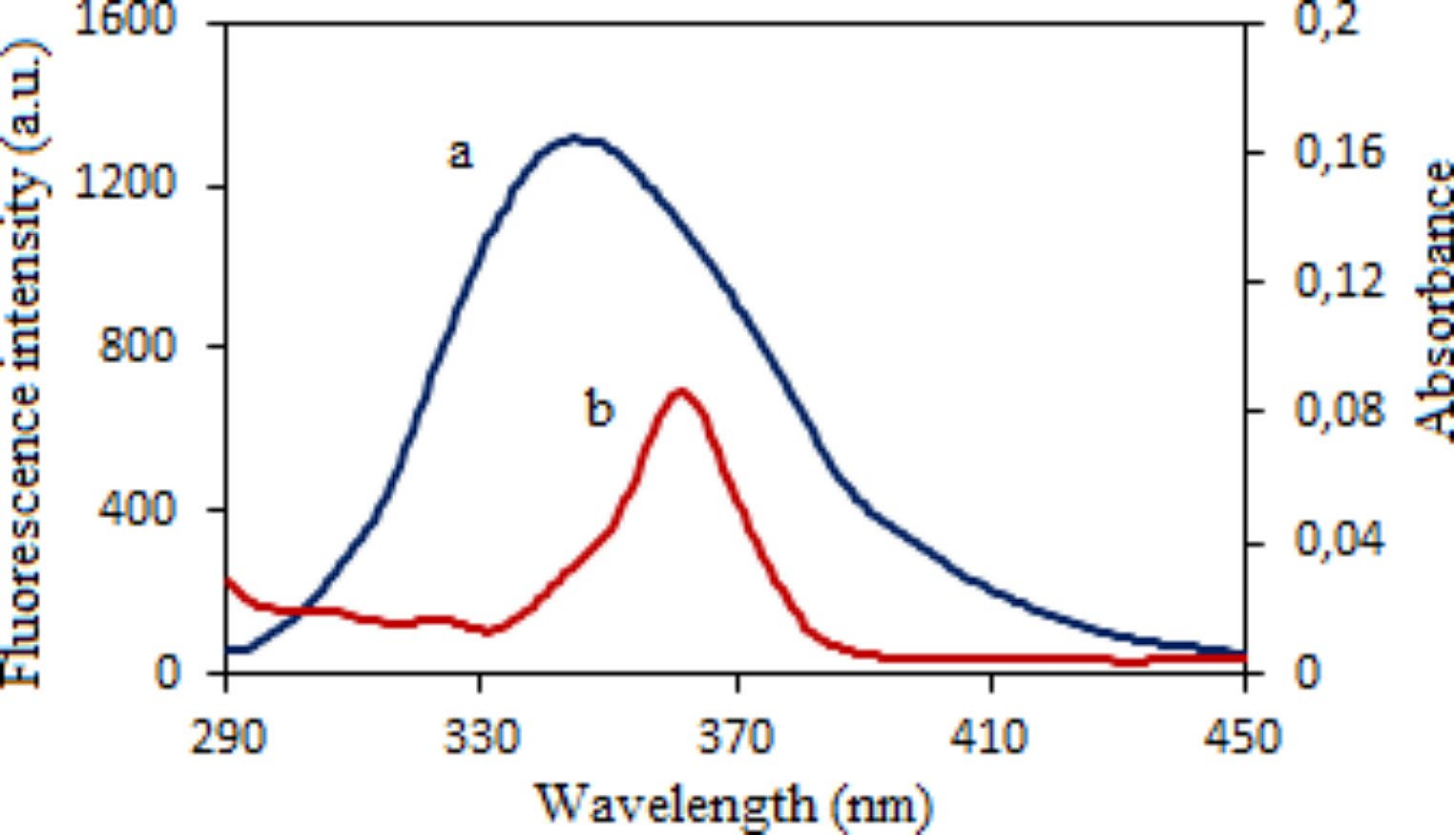
The overlap spectra of fluorescence **(a)** of 1.0 µM fibrinogen with absorption **(b)** of 1.0 µM vitB12

The overlap of UV-vis absorption spectrum of vitB12 with the fluorescence emission spectrum of Fib is shown in Fig. 4. According to the Förster’s non-radiative energy transfer theory [20, 21], the energy transfer can occur from the donor molecule (Fib) to the acceptor molecule (vitB12) and the distance between donor and acceptor can also determined using following equations;

5 $${\rm{E}}\,{\rm{ = }}\,{\rm{1}}\,{\rm{-}}\,{\rm{F/}}{{\rm{F}}_{\rm{0}}}\,{\rm{ = }}\,{{\rm{R}}_{\rm{0}}}^{\rm{6}}{\rm{/}}\left( {{{\rm{R}}_{\rm{0}}}^{\rm{6}}\,{\rm{ + }}\,{{\rm{r}}^{\rm{6}}}} \right)$$

6 $${{\rm{R}}_{\rm{0}}}^{\rm{6}}\,{\rm{ = }}\,{\rm{8}}{\rm{.79}}\,{\rm{ \times }}\,{\rm{1}}{{\rm{0}}^{{\rm{ - 25}}}}\,{\rm{(}}{{\rm{k}}^{\rm{2}}}{n^{{\rm{ - 4}}}}\,{{\rm{Q}}_{{\rm{D }}}}\,J{\rm{(\lambda ))}}$$

7 $$J{\rm{(\lambda )}}\,{\rm{ = }}\,{\rm{\Sigma }}\,{\rm{F(\lambda )}}\,{\rm{\varepsilon (\lambda )}}\,{{\rm{\lambda }}^{\rm{4}}}\,{\rm{\Delta \lambda /\Sigma }}\,{\rm{F}}\left( {\rm{\lambda }} \right)\,{\rm{\Delta \lambda }}$$

where F_0_ and F are the same as in Eq. (1), E and r are energy transfer efficiency and distance between donor and acceptor. R_0_ is the Förster critical distance at which 50% of the excitation energy is transferred to the acceptor. κ^2^, Q_D_ and n are the space factor of orientation, fluorescence quantum yield of the donor and refractive index of the working medium, respectively. *J*(λ) is the spectral overlap integral of the fluorescence emission spectrum of the donor and the absorption spectrum of the acceptor. Here, it obtained by integrating spectra for λ = 290–450 nm wavelength range in Fig. 4 according to Eq. (7). In calculations, using κ^2^ = 2/3, *n* = 1.336 and Q_D_ = 0.15 for fluorescence quantum yield of Trp residues of fibrinogen [5], the following parameters were obtained as *J*(λ) = 1.612 × 10^− 14^ M^− 1^cm^3^, E = 0.1066, *r* = 3.94 nm and R_0_ = 2.76 nm. The fact that r is less than 8 nm and 0.5 R0 < *r* < 1.5 R_0_ are evidence of non-radiative energy transfer from fibrinogen to vitB12.

### 3D-Fluorescence Measurements

**Fig. 5:**
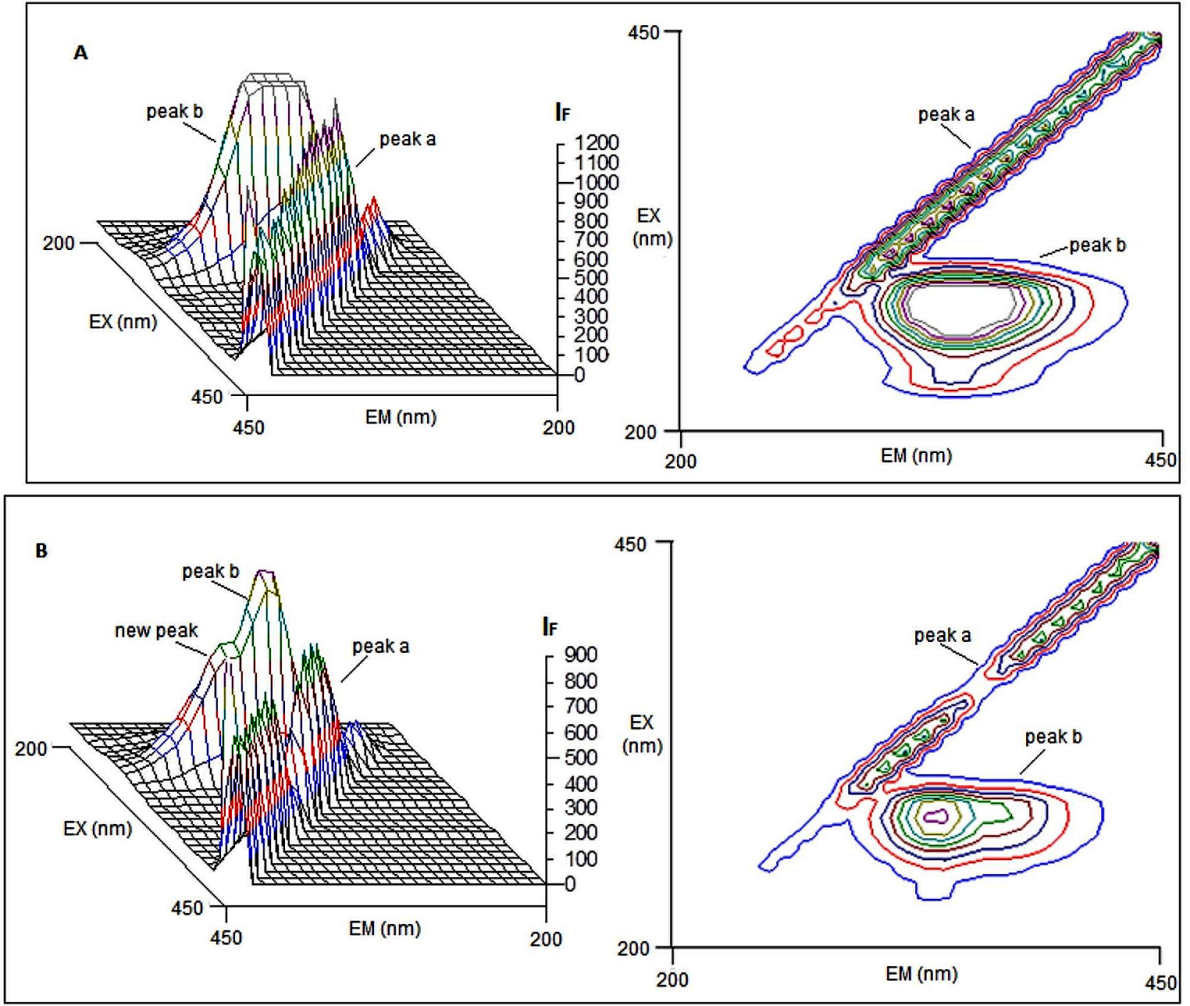
3D-fluorescence spectra (the left) and corresponding contour maps (the right) of fibrinogen alone (**A**) and vitB12-Fib complex (**B**). [Fib] = 1.0 µM; vitB12-Fib complex at [Fib] = 1.0 µM and [vitB12] = 7.8 µM; T: 298 K

The three-dimensional (3D) fluorescence technique is used in recent years for the conformational change of protein interacting with small molecules [22, 23]. The fluorescence spectra contains excitation wavelength, emission wavelength and fluorescence intensity at three axes. Figure 5 shows 3D-fluorescence spectra and the corresponding contour maps of fibrinogen alone (Fig. 5A) and vitB12-Fib binding system (Fig. 5B) while Table 2 summarizes the fluorescence characteristics of these spectra. The figures contains main two peaks; peak a refers to the Rayleigh scattering peak (where λ_ex_ = λ_em_) which is usually observed in the 3D-fluorescence studies. The strong peak b (λ_ex_/λ_em_ = 280/348 nm) shows the spectral characteristics of Trp and Tyr residues, involving π-π* transition in fibrinogen molecule [24]. In Fig. 5B, it was seen that the intensity of peak b clearly decreased with the addition of vitB12, while at the same time the formation of a new additional peak around 380 nm was presented [8]. The results show that a complex is formed between vitB12 and fibrinogen and there are changes in the peptide structure of the protein molecule.

**Table 2:** 3D-fluorescence spectral characteristics of fibrinogen and vitB12-Fib system

Peak	Fibrinogen			vitB12 Fibrinogen		
	Peak position	Stokes shift	IF	Peak position	Stokes shift	IF
	λ_ex_/λ_em_	Δλ(nm)	(a.u.)	λ_ex_/λ_em_	Δλ(nm)	(a.u.)
Peak a	230/230→450/450	0	1200	230/230→450/450	0	900
Peak b	280/348	68	1740	280/337	57	1320

### Determination of vitB12 by Fluorescence Quenching

The several instrumental methods have been developed for determination of vitB12, some of them containing the detection of Co(II) in vitamin structure; some of them containing vitB12 in food, phamaceuticals and biological materials. The common methods include such as chemiluminescence [25, 26], X-ray fluorescence [27], microbiological [28], radioisotopic assay [29], electrochemical analysis [30]. Here, we presented for determination of vitB12 by fluorescence quenching method. The Trp fluorescence of fibrinogen at fixed concentration (1.0 mM) was quenched regularly with increasing the amount of vitB12. The linear Stern-Volmer plot (F_o_/F = 0.8093 + 1.18 × 10^5^ [Q] with the correlation coefficient, R^2^ = 0.996) was used for the determining of vitB12 in the presence of fibrinogen at 298 K under the experimental conditions described above. The limit of detection (LOD) and limit of quantification (LOQ) of vitB12 were calculated using the formula 3*σ/m* and 10*σ/m*, respectively. *σ* is the standard deviation of the intercept and *m* is the slope of the calibration plot [31]. The replicate number of measurements is *n*. The LOD for vitB12 was obtained as 2.08 µM, which was consistent with the results given in the literature. Souza et all presented for vitB12 the LOD as 3.40 µM in electrochemical determination [30]. The obtained analytical data were listed in Table 3. To assess the precision and accuracy of our method, the relative standard deviation (RSD) was 4.28% as obtained from four replicate measurements of 7.80 µM of vitB12.

**Table 3:** The analytical data for determination of vitB12

Dynamic range of vitB12 (µM)	1.96 - 19.8
*σ* standard deviation of the intercept	0.0818
Slope of plot (m)	1.18 × 10^5^
Limit of detection (LOD) (µM)	2.08
Limit of quantification (LOQ) (µM)	6.92
Measurement number	7
RSD%	4.28

#### Absorption Studies

**Fig. 6:**
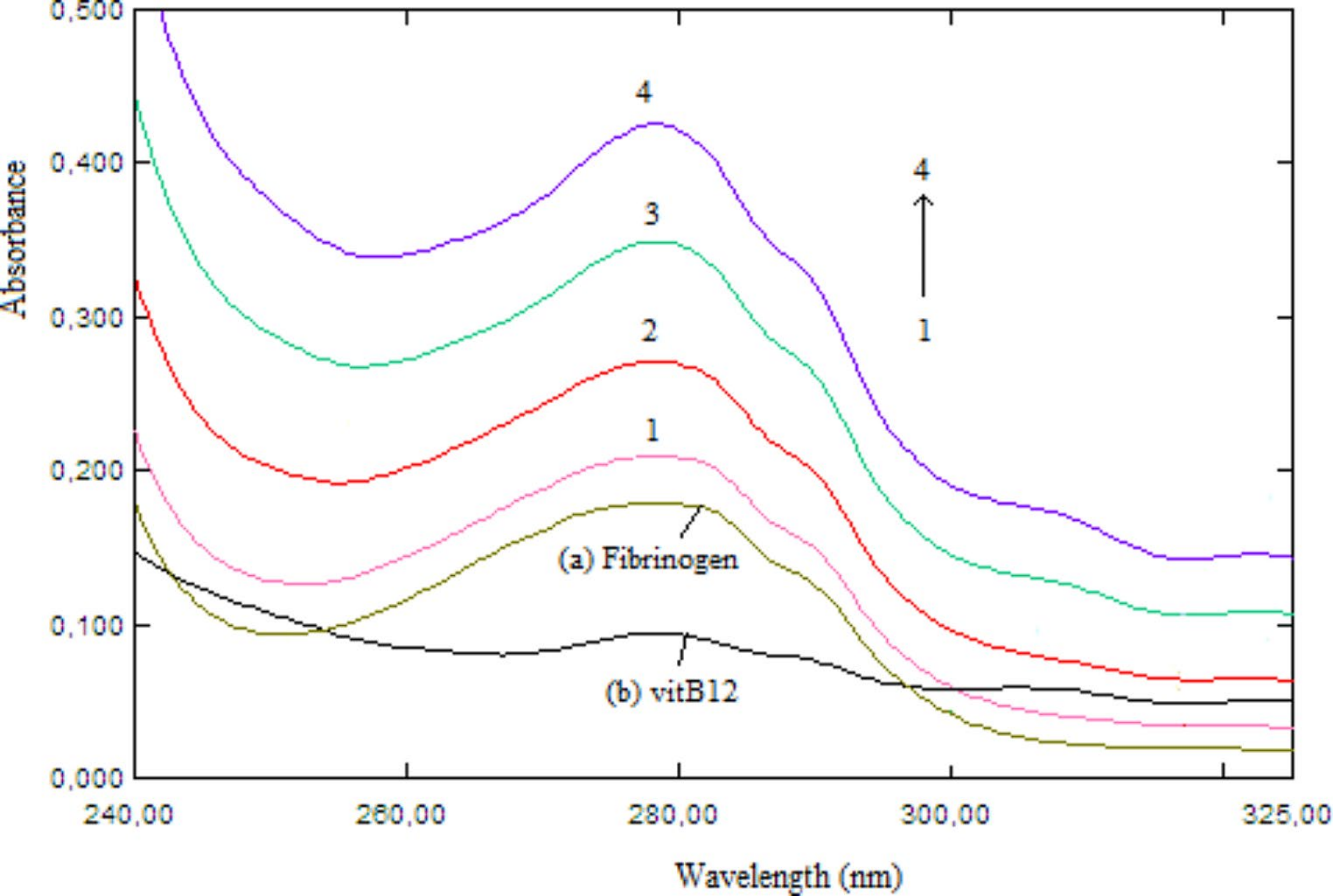
UV absorption spectra of fibrinogen in absence and presence of vitB12 at 298 K. Fibrinogen concentration fixed at 1.0 µM **(a)**, while that of vitB12 was varied from 2.0, 3.9, 6.0, 7.6 µM (1–4). **(b)** represents 10 µM free vitB12

Uv-vis absorption spectroscopy is a simple but effective technique in detecting complex formation between small molecule and protein. When a complex is formed between the species, changes of absorbance and position of band should occur [32]. Figure 6 UV absorption spectra of Fib (1.0 µM) having a peak at 280 nm in absence and presence of varying amount of vitB12 (0-7.6 µM). It can be seen the absorption band of Fib exhibited gradual increased and slightly blue shift (~ 3 nm) the increasing amount of vitB12. The hyperchromic effect with a small blue shift indicates a ground state complex formation between Fib and vitB12.

### Molecular Docking

**Fig. 7:**
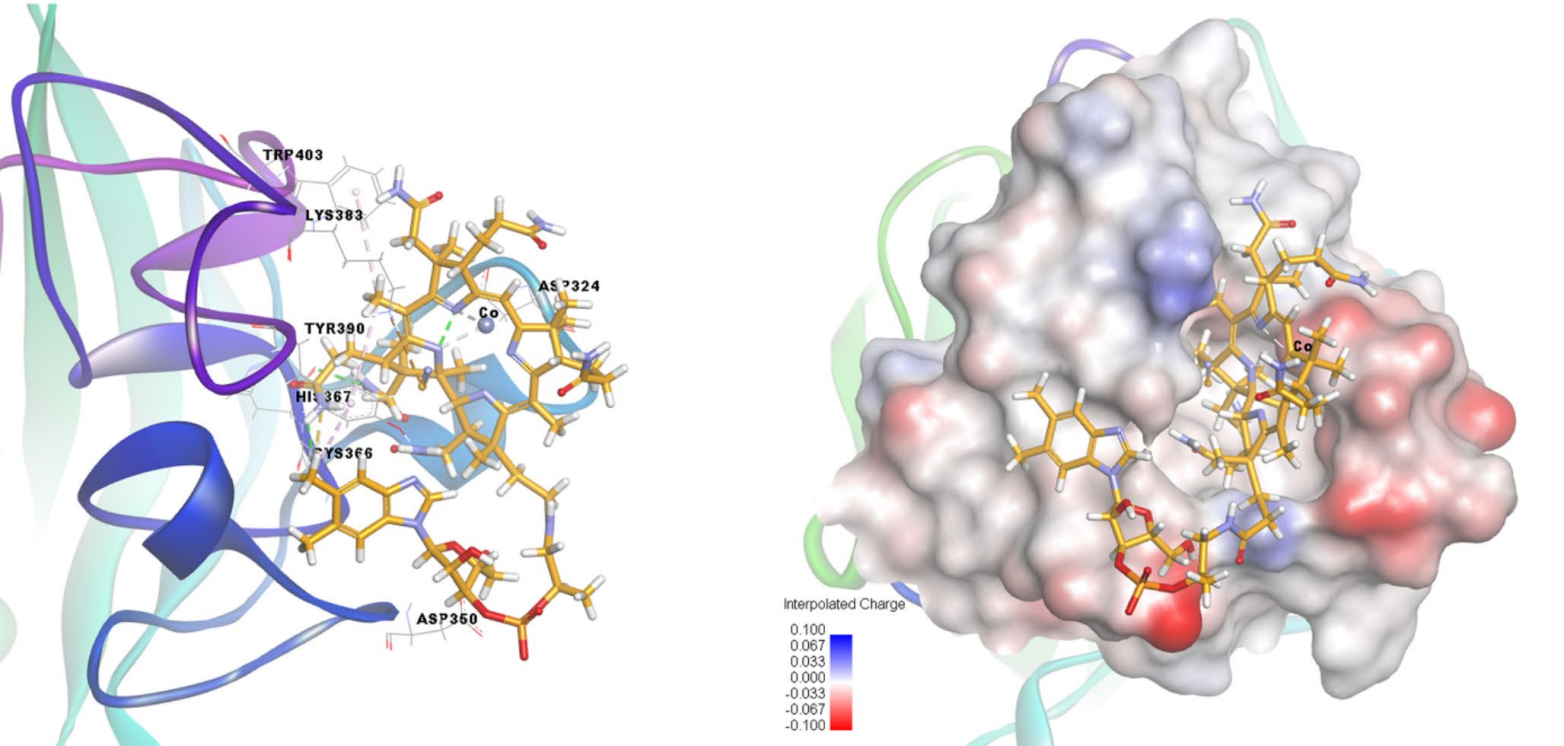
Docking conformation and interactions of vitB12 with fibrinogen

Molecular docking was performed for compound vitB12 against Fib. Docking results suggest that vitB12 has electrostatic, hydrogen bonding, metal and hydrophobic interactions with the target model. Figure 7 even shows that compound vitB12 forms one electrostatic interaction with Lys383 of Fib, four hydrogen bonds with residues Asp324, Asp350, His367, Cys366; one sulfur-nitrogen interaction with Cys366 amino acid and three hydrophobic interactions with residues Tyr390 and Trp403 in the binding site of Fib. At the same time, the interpolated charge surface of the formed complex exhibited the most stable and appropriate orientation with a binding energy value of -8.21 kcal/mol and RMSD value of 1.870 Å. Detailed interaction distances and binding types are tabulated in Table 4. According to Fig. 7, average intermolecular distance of vitB12 with Trp residues of Fib was calculated as 5.40 Å for three hyrophobic interactions from docking result. The binding distance (r) between Trp residues of fibrinogen (donor) and vitB12 (acceptor) molecules was calculated as 39.4 Å from experimantal FRET assays. The difference between the two results can be explained by the computational ommisions to match reality, rigidity of protein structure and absence of solvent molecules in computational calculations. The obtained short distance data between donor-acceptor pair indicates strong interaction of vitB12 with amino acid residues of Fib.

**Table 4:** Interactions types and distances of vitB12 with fibrinogen

Distance Å	Bonding	Binding site of target (Fibrinogen)	Binding site of ligand (B12)
5.2634	Electrostatic	A: LYS383:NZ	:B12:C69
3.0471	Hydrogen Bond	A: ASP324:OD2	:B12:N17
1.8368	Hydrogen Bond	A: ASP350:OD2	:B12:H173
3.0992	Hydrogen Bond	A: HIS367:O	:B12:H132
2.5435	Hydrogen Bond	A: CYS366:SG	:B12:H151
3.0833	Other	A: CYS366:SG	:B12:N24
5.4114	Hydrophobic	A: TYR390	:B12:C56
5.4625	Hydrophobic	A: TYR390	:B12:C93
5.3308	Hydrophobic	A: TRP403	:B12:C56

## Conclusion

In this study, the binding properties of vitaminB12 and fibrinogen system was explained by UV absorption and (steady state and 3D) fluorescence spectroscopies as well as molecular docking data. VitB12 has an ability to quench the intrinsic Trp residues of fibrinogen with static mechanism by forming the non-fluorescent complex. The positive values of ΔH (92.18 kJ/mol) and ΔS (433.5 J/molK) suggested that the binding of vitB12 could bind to the residues of fibrinogen mainly through hydrophobic forces with spontaneous entropy driven reactions. Molecular docking studies confirmed the existence of hydrophobic interaction. Additionally, docking results showed that hydrogen bonds were also effective in the binding process. The binding distance, r, between vitB12 and Fib was obtained as 3.94 nm accaording to Förster non-radiative energy transfer theory. The detection limit (LOD) and quantification limit (LOQ) of vitB12 using quenching method were calculated to be 2.08 µM and 6.92 µM in the presence of fibrinogen, respectively. This study which examines the binding affinity of fibrinogen, an important protein in blood clotting, and vitB12, an important vitamin for blood health, aims to shed light on the interaction studies of other blood proteins and vitamins. In the same time, theoretical application such as molecular docking investigations were divulged the interaction mechanism and orientations between vitB12 and fibrinojen at atomic level, is complementary and important part of the this research.

## Data Availability

No datasets were generated or analysed during the current study.
